# Cervical Thymic Cyst with parathyroid tissue – a diagnostic conundrum

**DOI:** 10.4322/acr.2021.361

**Published:** 2022-02-21

**Authors:** Deepika Gupta, Darwin Kaushal, Deepak Vedant, Rashim Sharma, Vikarn Vishwajeet, Poonam Abhay Elhence

**Affiliations:** 1 All India Institute of Medical Sciences, Department of Pathology and Lab Medicine, Jodhpur, Rajasthan, India; 2 All India Institute of Medical Sciences, Department of Otorhinolaryngology, Jodhpur, Rajasthan, India

**Keywords:** Cysts, Branchioma, Child Development, Pathology, Surgical

## Abstract

Cervical thymic cysts are relatively rare benign cystic lesions that tend to be diagnosed clinically as branchial cysts, which usually present as painless, enlarging neck masses. They can occur anywhere along the normal path of descent of thymic primordia from the angle of the mandible to the sternal notch, with mediastinal extension observed in approximately 50% of cases. They are usually seen in the first decade of life on the left side with a male predominance. Here we report a case of a 15-year-old boy who presented to the hospital with left-sided neck swelling for about 2 months. The neck’s contrast-enhanced computed tomography (CECT) revealed a large, well-defined cystic swelling in the left neck region, showing peripheral enhancement, seen from the submandibular region to the superior mediastinum extending into the retrosternal region. Direct fine needle aspiration (FNA) was done, which showed a benign lesion with inflammatory and cystic characteristics, leading to the possibility of a branchial cyst. The cyst was completely excised surgically. Histopathology showed a thymic cyst with parathyroid tissue. The presence of thymic tissue with Hassall’s corpuscles is essential for the diagnosis. Knowledge of the clinical presentation, cyto-histological findings, and differential diagnosis of cystic cervical lesions in the pediatric population is important to diagnose this rare entity. Hence, though uncommon, when one comes across a cystic cervical region mass in children, a diagnosis of cervical thymic cyst should be kept in mind. Nonetheless, a definitive diagnosis depends on imaging findings as well as intraoperative findings and histopathological examination.

## INTRODUCTION

Cystic neck swellings are usually related to developmental malformations. Anomalies of the lateral cervical region are distinctive to those of the midline anomalies. Cystic masses of the lateral cervical region occur due to developmental abnormalities involving the pharyngeal pouches as well as branchial clefts, which includes branchial cysts, lymphangiomas, and thymic cyst. In contrast, the midline anomalies occur due to abnormal closure of the midline or due to thyroid tissue remnants that persist after migration, which includes bronchogenic cysts, thyroglossal duct cysts and dermoid cysts.^1^ Cervical thymic cysts (CTCs) usually present during childhood and adolescence. About 1% of the cystic cervical lesions are found to be thymic cysts,^2^ and of these, about 0.3% occur congenitally in children.^3^ Two different theories have been postulated regarding the origin of these thymic cysts. One theory supports their origin from ectopic remnants of thymic tissue due to secondary degenerative changes in the Hassall’s corpuscles. The second theory favors the origin from cystic degeneration of persistent thymo-pharyngeal duct.^4^ The thymic tissue descends into the mediastinum during development. Cystic degeneration of these thymic tissue remnants may also lead to a thymic cyst.^5^ A correct preoperative diagnosis is rarely made because these cystic lesions are usually misdiagnosed as cystic lymphangioma, branchial cysts, and thyroglossal duct cyst, and hence, histopathology is required for confirmation.

This case report aims to present the observation along with a review of literature and evidence on the clinical features, diagnostic aspects, and management.

## CASE REPORT

**Figure1:**
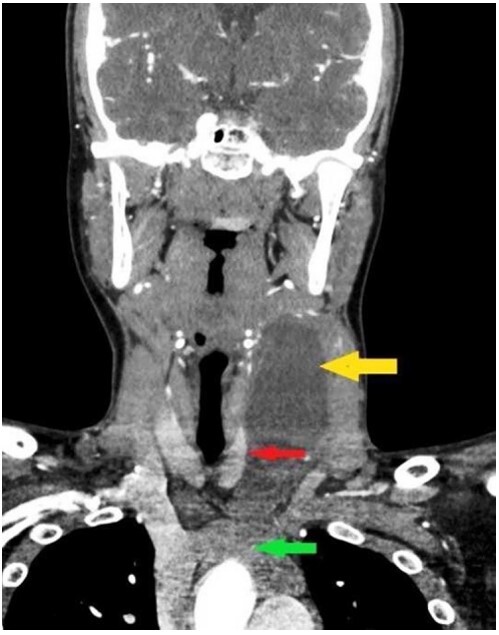
Neck CECT (coronal view) showing a large non-enhancing cyst (yellow arrow) compressing the thyroid lobe (red arrow) reaching to the superior mediastinum (green arrow at the arch of aorta).

A 15-year-old boy presented to our hospital with a left-sided neck swelling for about 2 months. The patient noticed the neck swelling incidentally, which initially measured about 3x3 cm and progressed over 2 months to reach the present size of 11x4cm. There was no history of neck pain, fever, weight loss, loss of appetite, trauma, or difficulty in eating. On examination, the swelling was non-tender, non-pulsatile, non-translucent, mobile more in the vertical direction than horizontal direction with overlying normal skin temperature. The swelling did not move with deglutition or protrusion of the tongue. The neck contrast-enhanced computed tomography (CECT) revealed a large, cystic, well-defined swelling in the left neck region. The swelling showed peripheral enhancement and was seen from the submandibular region to the superior mediastinum extending into the retrosternal region (Figure 1).

Direct FNA was performed using 23 gauge needle. 16ml of greenish-brown, clear to hazy fluid was aspirated, and the swelling apparently subsided after aspiration. The fluid was centrifuged, and post-centrifugation smears were subsequently stained with Papanicolaou, Hematoxylin and Eosin (H&E), and Leishman-Giemsa stains. Post-centrifugation smears were cellular and showed inflammatory infiltrate of cyst macrophages, lymphocytes, eosinophils, hemosiderophages, and occasional histiocytic giant cells. Few mature squamous epithelial cells, anucleate squames, proteinaceous debris, and numerous cholesterol crystals were noted in a fluidy proteinaceous background. No thyroid follicular epithelial cells were seen. The FNA was reported as a benign inflamed cystic lesion, and the possibility of an inflamed branchial cyst was given.

**Figure 2:**
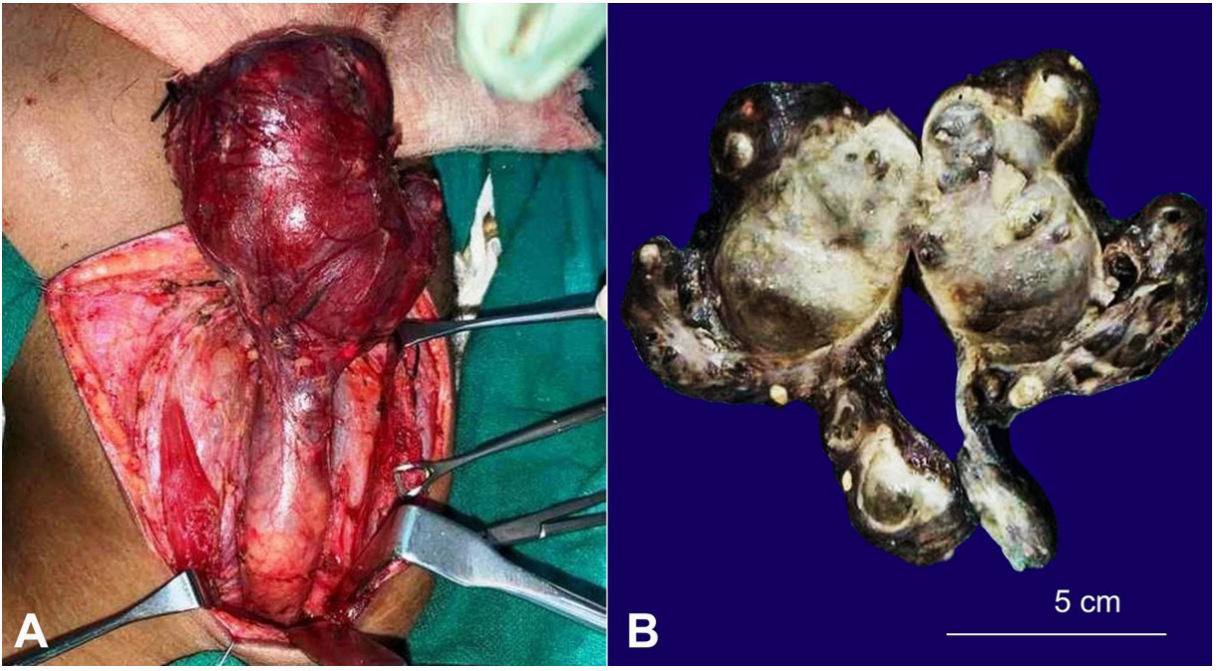
**A** – Intra-operative picture showing left cervical region cyst with tubular stalk extending to the mediastinum; **B** – Gross image, bisected cyst with tubular stalk.

Following the FNA diagnosis and imaging findings, the neck mass was resected (Figure 2A) and was sent for histopathological evaluation.

**Figure 3:**
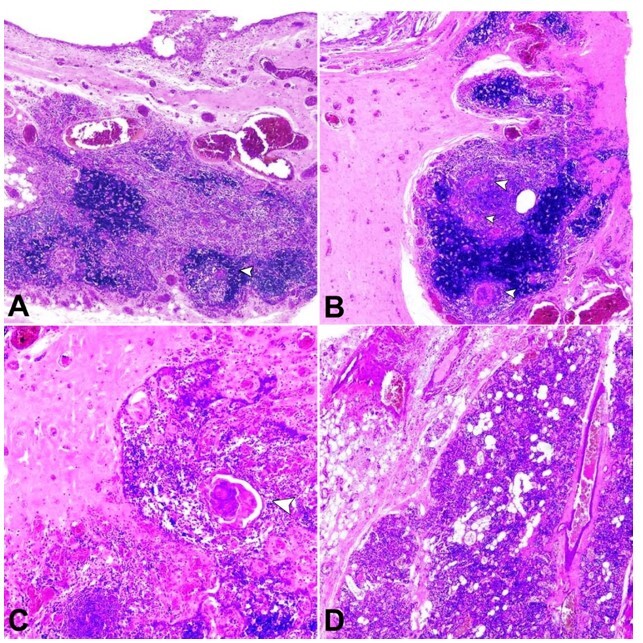
**A** – Cyst wall showing cyst lined by low cuboidal epithelium, sub-epithelial thymic tissue with Hassall’s corpuscles (arrowhead) and many congested vessels (H&E, 2X); **B** – Cyst wall containing cholesterol clefts, foreign body giant cells, sub-epithelial thymic tissue with Hassall’s corpuscles (arrowheads) and congested vessels (H&E, 2X); **C** – Cyst wall containing thymic tissue with Hassall’s corpuscles (arrowhead) (H&E, 4X); **D** – Cyst wall with parathyroid tissue (H&E, 2).

An intact brownish-grey cystic mass, 11.5cm in length, 80 grams in weight, was received with an elongated tubular projection at one end. The cystic mass measured 7x5x2.8cm, and the elongated tubular projection measured 4.5x2.2x2.6cm. Sectioning yielded about 14ml of turbid greenish fluid. The cut surface showed a large unilocular cyst with wall thickness ranging from 0.3 to 0.6 cm. The elongated tubular projection showed cyst with sieve-like areas (Figure 2B). Histopathological evaluation showed a cyst with fibrocollagenous wall lined by attenuated to ulcerated and focal cuboidal to squamous epithelium, many cholesterol clefts with associated foreign body multinucleated giant cells (Figure 3A, B). Nests of thymic tissue and Hassall’s corpuscles were noted (Figure 3C). Parathyroid tissue was noted (Figure 3D). Many perivascular lymphoid aggregates were seen. A final diagnosis of the cervical thymic cyst with parathyroid tissue was rendered. The postoperative period was uneventful without any complications. The patient is asymptomatic more than one year and a half after surgery with a well-healed scar.

## DISCUSSION

Cervical thymic cysts are rare neck lesions with approximately less than 120 cases documented in the literature.^4^ These cysts are usually asymptomatic and have an indolent growth rate, eventually reaching a large size. They occur in the region between the angle of the jaw and the sternum. Due to a larger thymus in children, most of the patients tend to present in the pre-pubertal age, with cases being slightly more frequent in males.^6^ About half of these cystic swellings are seen in continuity with the normally located mediastinal thymus,^7^ and 60-70% of these cysts have been noted in the left neck region.^8^ The present case was seen in the left side of the neck in a 15-year-old boy, and no continuity with the mediastinal thymus was evident.

The thymus originates from the third and fourth pharyngeal pouches and forms the main lymphoid organ during infancy and in children. The thymic primordia emanate from the pyriform sinus, passes through the lateral part of the thyroid gland and descends to the mediastinum. The thymic tissue can be found along this path of descent of the thymic primordia, due to which cervical thymic cysts can be found in the region from the mandible to the sternum.^9^ Many theories of the origin of thymic cysts are propounded, but two main theories regarding pathogenesis are there. One is acquired progressive cystic degeneration in Hassall’s corpuscles and the thymus’ epithelial reticulum, giving rise to an acquired multilocular thymic cyst.^10^ A second etiology favors cystic change in persistent unincorporated remnants of the thymopharyngeal duct giving rise to a congenital unilocular thymic cyst. This theory is favored due to the frequent association of cervical thymic cysts with other endocrine glands like parathyroid or thyroid.^11^ As both the thymus and the parathyroid glands are derived from 3rd and 4th pharyngeal pouches, the wall of the thymic cyst may contain parathyroid tissue.^12^ Despite common developmental origin, coexisting parathyroid tissue has been documented in only five reports in children besides the current case (Table 1).^4^^,^ 8 ^,^ 12 ^,^ 13 ^,^ 14 Few cases of rare thymic cysts with coexisting parathyroid tissue have been seen in adults.^5^

**Table 1:** Reported cases of cervical thymic cyst with coexisting parathyroid tissue in children

	**Author**	**Year**	**Age**	**Gender**	**Laterality**	**Coexisting parathyroid tissue**
1	Present case	2019	15 y	M	Left	Seen
2	Jindal A^12^	2016	14 y	M	Left	Seen
3	Jaiswal AA^8^	2014	8 y	M	Left	Seen
4	Daneshbod Y^13^	2006	6 y	M	Left	Seen
5	Berenos-Riley L^14^	2005	7 y	M	Left	Seen
6	Nguyen Q^4^	1996	8 d	F	Left	Not seen
			12d	M	Left	Not seen
			5 y	M	Right	Not seen
			9 y	F	Left	Seen

The thymic cysts are mostly elongated and range from 1cm to 26cm in diameter.^4^^,^ 8 ^,^ 15 The cyst contents may have varied gross appearance from clear, serous to brownish fluid. Histopathological evaluation of excised specimens is the only definitive means for diagnosing a thymic cyst.^15^ The presence of thymic tissue with Hassall’s corpuscles is essential for diagnosing of a cervical thymic cyst.^15^ The periphery of the cyst wall may show parathyroid gland tissue.^13^

A preoperative diagnosis is rarely formed, and the mass is removed by surgery which is the mainstay of treatment, which is both diagnostic as well as therapeutic. In 1901, Pollosson and Piery^16^ first attempted partial surgical excision. However, Hyde et al.^15^ were the first to report successful removal of a cervical thymic cyst in 1944. Documentation of the thymus in the mediastinum is mandatory before the ectopic cystic thymic tissue is removed to avoid immune status impairment later in life.

Hsieh et al.^3^ analyzed 331 patients with cervical cystic masses below 18 years of age and found slight male preponderance with ratio of 1.6:1. Thyroglossal duct cyst (181 patients) was the most common congenital neck cyst, which was followed by cystic hygromas in 93 patients, 54 patients with branchial cleft cysts and 3 patients with bronchogenic cysts. Nine cases could not be classified. Only a single case of thymic cyst was seen. Sturm-O'Brien et al.^17^ carried out a retrospective analysis of cervical thymic region anomalies over a span of 25 years and found the mean age of presentation to be 5.6 years. Thymic cysts were much more common in males with a ratio of 5.5:1. They found the computed tomography (CT) scan to be the most reliable modality in giving an accurate diagnosis of the thymic cysts, and ultrasound-guided biopsy provided correct tissue diagnosis of ectopic thymic tissue.

The main entity to be differentiated is a branchial cyst that originates from the thymopharyngeal tract of the second pouch and lymphangioma. The patient's age and location of the cyst help in differentiating. The branchial cysts are located superficial and lateral to the internal jugular vein and common carotid artery, and ends in the superior tonsillar fossa and lymphangiomas are found in the posterior triangle of the neck. In contrast, thymic cysts pass posterior to carotid bifurcation, situated in close association with the carotid sheath and terminates in the pyriform fossa. Also, branchial cleft cysts rarely extend to the clavicle while thymic cysts tend to be larger, extending toward the anterosuperior mediastinum. Also, branchial cleft cysts are seen in young adults around the third decade of life, while thymic cysts tend to be pre-pubertal usually. Different imaging modalities are also helpful. Magnetic Resonance Imaging (MRI) is considered superior to ultrasonography (USG) and CT scan owing to its multiplanar capability and superior contrast resolution. On CT scan, the thymic cyst may appear as uni or multiloculated, hypoattenuating cystic mass present alongside the carotid space. No contrast enhancement is seen except in cases with solid components, which might represent aberrant thymic tissue, parathyroid tissue, or lymphoid tissue.^18^ On Magnetic Resonance Imaging (MRI), they are usually hypointense on T1 and hyperintense on T2. Branchial cleft cysts usually are well-circumscribed, homogeneous unilocular cysts, whereas lymphangiomas are multilocular, predominantly cystic with septae of variable thickness.^19^

The differentials of cervical cystic neck masses are dermoid cyst, epidermoid cyst, thyroglossal duct cyst, laryngocele, hemangioma, bronchogenic cyst, thyroid lesions, cervical lymphadenopathy, rarely parathyroid cysts and malignancies.^5^^,^ 7 A remote possibility of malignant neoplastic lesions arising from these cystic lesions is very rare which usually arise in association with heterotopic tissue and malignant cystic metastasis like papillary thyroid carcinoma should be suspected if heterogenous cystic and solid mass is found clinically and radiologically.^19^ Positron-emission tomography (PET) may be used to identify orthotopic thymic tissue within the anterior mediastinum or ectopic location along the course of its embryological development. Orthotopic thymus appears as bi-lobed with mild FDG avidity.^20^ Using radio-immunoassay, cystic fluid aspirate may be used to estimate the concentration of thyroglobulin and parathormone to rule out thyroglossal cyst and parathyroid cyst, respectively.

## CONCLUSION

Cervical thymic cysts are rare but should be borne in mind in the differentials of cystic neck swellings, especially in the prepubertal age group. Definitive diagnosis depends on imaging modality, intraoperative surgical findings, and histopathological examination. A correct diagnosis can be rendered if the pathologists and clinicians are acquainted with this entity.
